# Efficacy of Flash Glucose Monitoring in Type 1 and Type 2 Diabetes: A Systematic Review and Meta-Analysis of Randomised Controlled Trials

**DOI:** 10.3389/fcdhc.2022.849725

**Published:** 2022-02-22

**Authors:** Bonnie Liang, Digsu N. Koye, Mariam Hachem, Neda Zafari, Sabine Braat, Elif I. Ekinci

**Affiliations:** ^1^Department of Medicine, Austin Health, Melbourne Medical School, University of Melbourne, Heidelberg, VIC, Australia; ^2^Department of Medicine, Royal Melbourne Hospital, The University of Melbourne, Melbourne, VIC, Australia; ^3^Centre for Epidemiology and Biostatistics, Melbourne School of Population and Global Health, The University of Melbourne, Melbourne, VIC, Australia; ^4^Department of Endocrinology, Austin Health, Heidelberg, VIC, Australia

**Keywords:** flash glucose monitoring, self-monitoring blood glucose, diabetes mellitus, glycated haemoglobin A1c, hyperglycaemia, hypoglycaemia, meta-analysis, systematic review

## Abstract

**Objective:**

Flash glucose monitoring (FlashGM) is a sensor-based technology that displays glucose readings and trends to people with diabetes. In this meta-analysis, we assessed the effect of FlashGM on glycaemic outcomes including HbA_1c_, time in range, frequency of hypoglycaemic episodes and time in hypo/hyperglycaemia compared to self-monitoring of blood glucose, using data from randomised controlled trials.

**Methods:**

A systematic search was conducted on MEDLINE, EMBASE and CENTRAL for articles published between 2014 and 2021. We selected randomised controlled trials comparing flash glucose monitoring to self-monitoring of blood glucose that reported change in HbA_1c_ and at least one other glycaemic outcome in adults with type 1 or type 2 diabetes. Two independent reviewers extracted data from each study using a piloted form. Meta-analyses using a random-effects model was conducted to obtain a pooled estimate of the treatment effect. Heterogeneity was assessed using forest plots and the I^2^ statistic.

**Results:**

We identified 5 randomised controlled trials lasting 10 – 24 weeks and involving 719 participants. Flash glucose monitoring did not lead to a significant reduction in HbA_1c_. However, it resulted in increased time in range (mean difference 1.16 hr, 95% CI 0.13 to 2.19, I^2^ = 71.7%) and decreased frequency of hypoglycaemic episodes (mean difference -0.28 episodes per 24 hours, 95% CI -0.53 to -0.04, I^2^ = 71.4%).

**Conclusions:**

Flash glucose monitoring did not lead to a significant reduction in HbA_1c_ compared to self-monitoring of blood glucose, however, it improved glycaemic management through increased time in range and decreased frequency of hypoglycaemic episodes.

**Systematic Review Registration:**

https://www.crd.york.ac.uk/prospero/, identifier PROSPERO (CRD42020165688).

## Introduction

Flash glucose monitoring (FlashGM) technology has revolutionised diabetes management. Its rising popularity has driven a need to assess its impact on key markers of glycaemic management. Hitting glycaemic targets is key to the success of diabetes management and yet, up to 92% of people with diabetes fail to achieve these targets (1). As a result, these individuals are susceptible to micro and macrovascular complications of diabetes, as well as excessive morbidity and increased risk of death (2–4). FlashGM is a sensor-based technology that displays current glucose levels, glucose readings from the past 8 hours and trend arrows (5). Unlike self-monitoring of blood glucose (SMBG), FlashGM produces an ambulatory glucose profile that displays key trends in hypo-, normo- and hyperglycaemia (5). It contributes to clinical care by providing clear, comprehensive glucose data with minimal inconvenience. Such information can be used to guide patient choice, clinical practice and future reimbursement of FlashGM (6).

Previous systematic reviews on FlashGM have reported inconsistent findings (7–9). They have all included observational studies without a control group as a comparator. To minimise bias and confounding factors, we examined randomised controlled trials (RCTs) to assess the effects of FlashGM on glycaemic management. Current literature largely focuses on HbA_1c_ as the main metric of treatment efficacy (8, 9). However, we recognise that glycaemic management has multiple dimensions including time in range, frequency of hypoglycaemic episodes and time in hypo/hyperglycaemia. Less time in range is associated with the increased risk of microvascular complications (10, 11) and hypoglycaemia is linked to life-threatening outcomes including neurocognitive dysfunction and cardiovascular dysfunction (12, 13). To ensure a comprehensive analysis of FlashGM, we assessed these glycaemic outcomes in addition to change in HbA_1c_.

The aims of this meta-analysis were therefore to assess the effect of FlashGM compared to SMBG on HbA_1c_, time in range (3.9 – 10.0mmol/L), time in hypoglycaemia (<3.9mmol/L), occurrence of hypoglycaemic episodes and time in hyperglycaemia (>10mmol/L) over a span of 10 to 24 weeks. Our primary hypothesis was that FlashGM leads to reduced HbA_1c_ in individuals with type 1 and 2 diabetes. Our secondary hypotheses were that FlashGM leads to: (i) increase in time in range (3.9 – 10mmol/L), (ii) reduction in time in hypoglycaemia (<3.9mmol/L), (iii) reduction in frequency of hypoglycaemic episodes and (iv) reduction in time in hyperglycaemia (>10mmol/L).

## Materials and Methods

This systematic review was registered in PROSPERO (CRD42020165688) and performed in accordance with the Preferred Reporting Items for Systematic Reviews and Meta-Analyses statement (14) (**Supplementary Figure 1**).

### Data Sources and Searches

A literature search was conducted on relevant databases including MEDLINE, EMBASE and the Cochrane Central Register of Controlled Trials (CENTRAL). The reference lists of retrieved studies were assessed for further relevant studies. With the help of an expert librarian, we developed a search strategy from Medical Subject Headings (MeSH) and text words related to diabetes, flash glucose monitoring and blood glucose from 1^st^ January 2014 until 13^th^ September 2021 (**Supplementary Figure 2**). The Cochrane Highly Sensitive Search Strategy was used to restrict the search to randomised controlled trials.

### Study Selection

Using Covidence software, two review authors (BL and MH) independently screened titles and abstracts for relevant studies. Covidence was used to exclude the duplicates and the remaining studies were assessed for eligibility by predetermined selection criteria. This was defined as: 1) participants aged ≥ 18 years, 2) type 1 or 2 diabetes, 3) use of FlashGM device as one of the intervention groups, 4) use of SMBG in control group, 5) report of HbA_1c_ (%) and at least one other glycaemic outcome such as time in range (hours or percentage), time in hypo/hyperglycaemia (minutes or percentage) or frequency of hypoglycaemic events per 24 hours, 6) at least 10 weeks duration of intervention and 7) randomised controlled trials. A hypoglycaemic episode was defined by glucose readings below 3.9 mmol/L for at least 15 minutes (15). The end of the episode was defined by readings above 3.9 mmol/L for 15 minutes (15). Studies that blinded participants in the intervention group to sensor glucose data were excluded because it is not reflective of FlashGM use in clinical practice. Access to glucose readings facilitates self-management behaviours as individuals may alter food intake, exercise or medication according to their glucose level (16–18). The influence of FlashGM on behaviour is key to assessing its efficacy in glycaemic management (16). We also excluded studies that used results from other trials and did not have their own original data. Since FlashGM was introduced in 2014, we only included studies published from 2014 onwards. Studies were not excluded on the basis of publication status or on the basis of language.

### Data Extraction and Quality Assessment

Studies that met the eligibility criteria were retrieved for full-text assessment. The data from included studies were extracted independently by two review authors (BL and MH) using a piloted form. Results were compared for accuracy and any differences were resolved by consensus. The following data was extracted from each study: 1) author(s) and publication year 2) study population 3) location of study 4) participants’ baseline characteristics such as age, diabetes type, type of treatment regimen and initial HbA_1c_ 5) duration of study 6) glycaemic endpoints including HbA_1c_ (mmol/mol), time in range (hours) and time in hypo/hyperglycaemia (hours) or frequency of hypoglycaemic events depending on availability of data and 7) mean difference and standard error of extracted glycaemic outcomes. The corresponding authors of four studies were contacted *via* email for missing data but no further information on desired outcomes was provided.

The Cochrane risk-of-bias tool (RoB 2) for randomised trials was used to assess the design, conduction and reporting of included studies. Two review authors (BL and MH) independently conducted the assessment, compared results and resolved differences by consensus. Bias was judged based on five domains: randomisation process, deviations from intended interventions, missing outcome data, measurement of outcome and selection of reported result. Each domain required answers to signalling questions to evaluate the potential risk of bias. The level of bias in each of the five domains was summarised to generate the overall risk of bias and classified as having low bias, high bias, or some concerns regarding bias.

### Data Synthesis and Analysis

Changes from baseline in HbA_1c_, time in range, time in hypo/hyperglycaemia and number of hypoglycaemic episodes per 24 hours were analysed as continuous variables using mean difference between groups and standard error as summary measures. When variability was reported in confidence intervals (CI), standard error (SE) was estimated using the following formula: SE = (upper limit – lower limit)/3.92. A random effects meta-analysis was conducted and effect sizes were reported in the form of mean difference and 95% confidence interval. The I^2^ statistic was used to quantify the percentage of variability in effect size that was due to heterogeneity. In this meta-analysis, low heterogeneity was defined as 40% or less, moderate heterogeneity was between 30% and 60%, substantial heterogeneity was 50% to 90% (19). For all outcomes, we pre-planned subgroup analyses according to type 1 and type 2 diabetes. However, only one study focused exclusively on type 1 diabetes, thereby precluding subgroup analysis. It should also be noted that time in range and time in hypo/hyperglycaemia were meta-analysed and reported in hours as hours was the unit originally reported in the included as publications. All statistical analyses were performed using STATA, version 16, (STATA, College Station, Texas).

## Results

### Search Results

**Figure 1:**
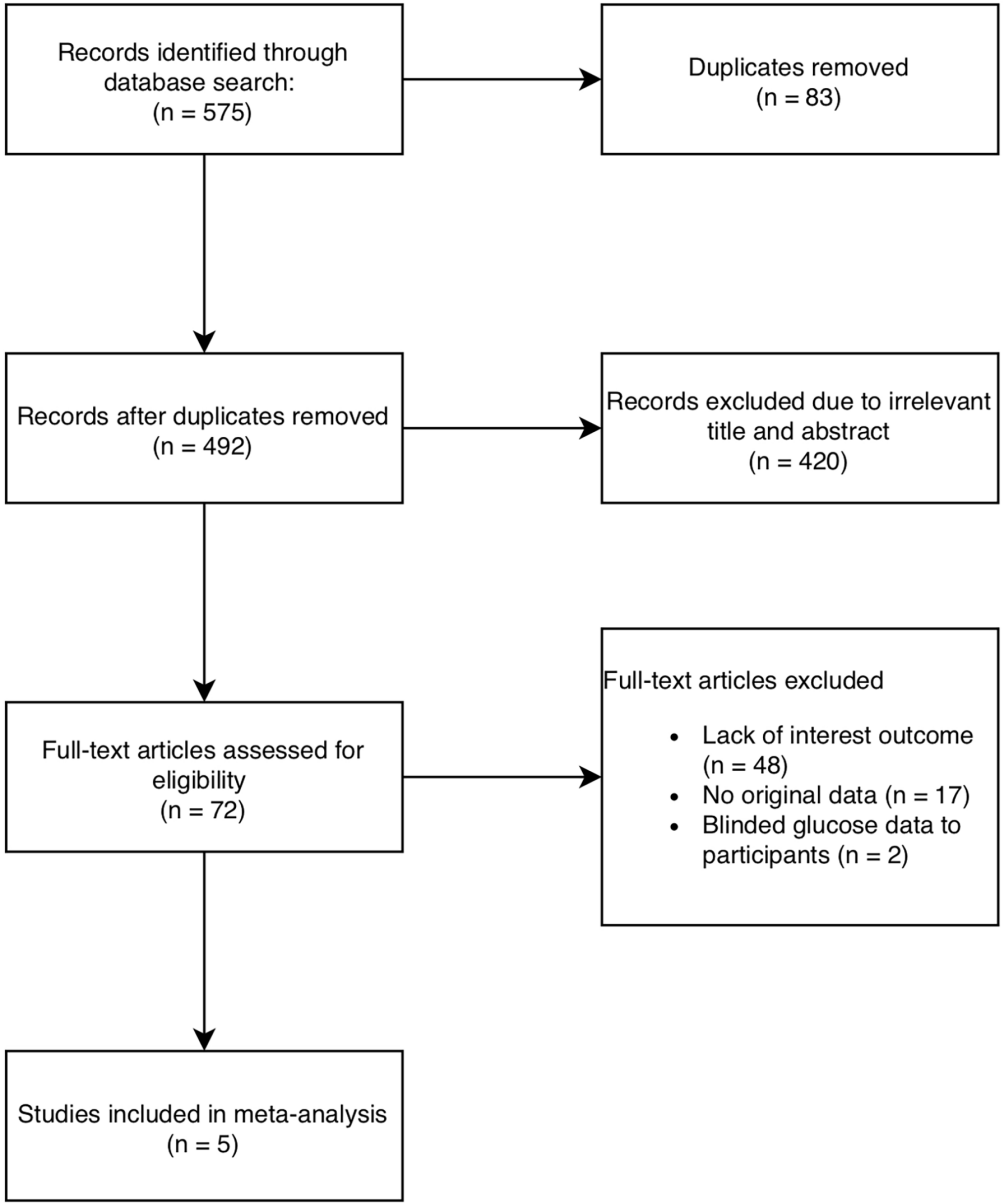
Flowchart of study diagram according to PRISMA guidelines.

Five hundred and seventy-five articles were identified using MEDLINE, EMBASE and CENTRAL and 492 records remained after duplicates were removed. Five RCTs including a total of 719 participants (400 in the intervention group and 319 in the control group), were included in the meta-analysis (20–24) (**Figure 1**).

### Study and Patient Characteristics

**Table 1** shows the characteristics of the included RCTs with baseline characteristics of participants. The trials were published between 2016 and 2020 and recruited participants from Europe, Australia, Japan and Israel. All studies had an open-label design and only included adult participants. All had a duration of 24 weeks except for one, which was 10 weeks long (24). Three studies were conducted on participants on insulin only (20, 21, 24), one study involved participants on both insulin and oral glucose lowering medications (22) and one study was on participants using oral glucose lowering medications only (23). One study was undertaken in people with type 1 diabetes (20), three studies focused on participants with type 2 diabetes (21, 23, 24) and one study assessed both individuals with type 1 and type 2 diabetes (22). Mean patient age ranged from 40.2 to 67.6 years and mean baseline HbA1c levels (%) (SD) ranged from 50.6mmol/mol (6.78%) (0.64) to 72.1mmol/mol (8.75%) (0.98).

**Table 1:** Characteristics of studies included in the meta-analysis.

First author, year	Study duration (weeks)	Diabetes type	N Intervention/control	Mean age intervention/control, years	Baseline HbA1c intervention/control, mmol/mol (%)	Diabetes regimen	Mean number of sensor scans per day	Primary outcome
Bolinder et al., 2016 (20)	24	1	119/120	40.2/45.0	51/51 (6.79/6.78)	CSII, MDI	15.1 (6.9)*	Difference in time in hypoglycaemia at 24 weeks
Davis et al., 2019 (22)	24	1 & 2	30/25	55.3/51.9	63/62 (7.9/7.8)	CSII, MDI & oral hypoglycaemics	NR	Incidence of severe hypoglycaemia requiring second party assistance
Haak et al., 2017 (21)	24	2	149/75	59.0/59.5	71/72 (8.65/8.75)	CSII, MDI	8.3 (4.4)*	Difference in adjusted means of HbA1c in intervention vs control at 24 weeks
Wada et al., 2020 (23)	24	2	49/51	58.1/58.7	62/62 (7.83/7.84)	Hypoglycaemic agents	NR	Difference in HbA1c from start to end of study at 24 weeks
Yaron et al., 2019 (24)	10	2	53/48	67.6/65.9	71/68 (8.68/8.34)	MDI	NR	Treatment satisfaction after 10 weeks

CSII, continuous subcutaneous insulin infusion; MDI, multiple daily insulin injection; NR, not reported; *data in parentheses are standard deviation.

### Risk of Bias

According to the Cochrane risk-of-bias tool, the risk of bias was evaluated as low in all studies except for one, which had a randomisation process that raised concerns (21) (**Supplementary Figure 3**). The lack of blinding between participants and personnel was also a potential source of bias. However, it is impractical to blind FlashGM usage as the glucose data informs personal choices regarding self-management (16–18). Blinding FlashGM data would not reflect real life settings and would impinge on the generalisability of our results.

### Change in HbA_1c_

**Figure 2:**
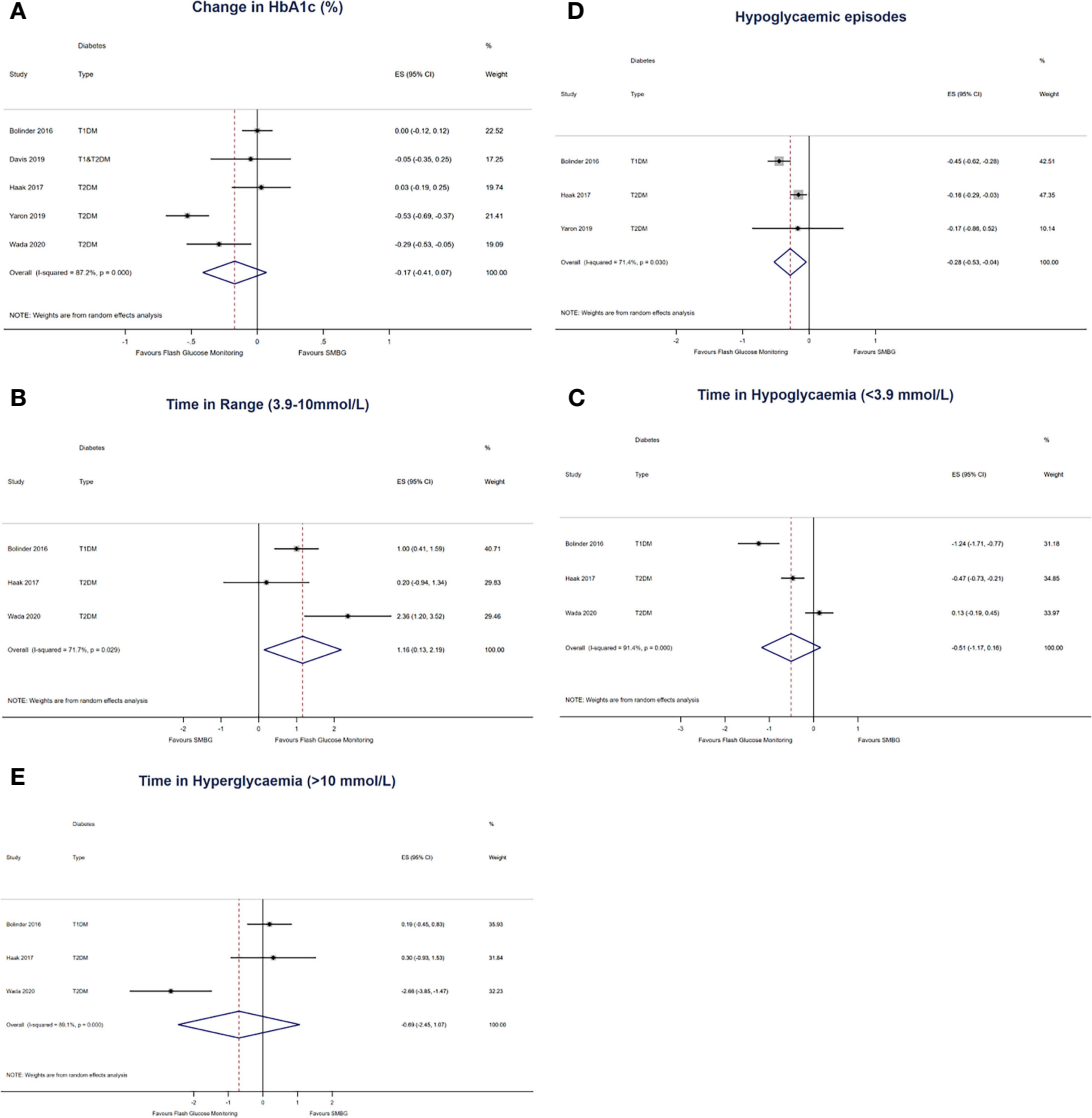
Forest plots of the effect size of **(A)** first-row left: change in HbA1c from baseline to last follow-up, expressed in % in all 5 comparisons. **(B)** first-row right: change in time in range (3.9 – 10mmol/L) per 24 hours, expressed in hours **(C)** second-row left: change in time in hypoglycaemia (<3.9mmol/L) per 24 hours, expressed in hours **(D)** second-row right: change in number of hypoglycaemic episodes per 24 hours **(E)** third-row left: change in hyperglycaemia (>10mmol/L), expressed in hours. The results are expressed as mean differences, compared to participants that used SMBG.

Five studies with 719 participants (400 in the intervention group and 319 in the control group) were pooled for the primary outcome of change in HbA_1c_. There was no statistically significant decrease in HbA_1c_ at endpoint in the FlashGM group compared to SMBG (mean difference: -0.17%, 95% CI -0.41 to 0.07, *p* = 0.164). Studies included for analysis had high heterogeneity (*I^2^* = 87.2%, *P <*0.001) (**Figure 2A**).

### Time in Range

Three studies with 563 participants (317 in the intervention group and 246 in the control group) were pooled for the outcome of time in range (20, 21, 23). Compared to SMBG, FlashGM was associated with a statistically significant increase in time spent in target glucose range (mean difference: 1.16 hr, 95% CI 0.13 to 2.19, *p* = 0.027). There was substantial heterogeneity between comparisons (*I^2^* = 71.7%, *p* = 0.029) (**Figure 2B**).

### Time in Hypoglycaemia

For this outcome, three studies with 563 participants (317 in the intervention group and 246 in the control group) were included (20, 21, 23). There was no statistically significant decrease in time spent in hypoglycaemia in the FlashGM group compared to SMBG (mean difference: -0.51 hr, 95% CI -1.17 to 0.16, *p* = 0.137). Overall, there was high heterogeneity between studies that assessed time in hypoglycaemia (*I^2^* = 91.4%, *P <*0.001) (**Figure 2C**).

### Hypoglycaemic Episodes

The outcome of frequency of hypoglycaemic episodes per 24 hours was based on data from three studies with 564 participants (321 in the intervention group and 243 in the control group) (20, 21, 24). Overall analysis of FlashGM showed a decrease in frequency of hypoglycaemic episodes per 24 hours compared to the control (mean difference: -0.28 episodes per 24 hours, 95% CI -0.53 to -0.04, *p* = 0.022). Heterogeneity between comparisons was substantial (*I^2^* = 71.4%, *p* = 0.030) (**Figure 2D**).

### Time in Hyperglycaemia

Three studies with 563 participants (317 in the intervention group and 246 in the control group) were pooled for the outcome of time in hyperglycaemia (20, 21, 23). There was no difference between time in hyperglycaemia in the FlashGM group and the control group (mean difference: -0.69 hr, 95% CI -2.45 to 1.07, *p* = 0.440). There was high heterogeneity between the comparisons (*I^2^* = 89.1%, *P <*0.001) (**Figure 2E**).

## Discussion

### Main Findings

In this meta-analysis, we found that the use of FlashGM did not result in a significant reduction in HbA_1c_, however, compared to SMBG, FlashGM resulted in improvements in two key glycaemic markers: time in range and frequency of hypoglycaemic episodes per 24 hours. The use of FlashGM led to a 1.16 hour per day increase in time in range and a 0.28 reduction in number of hypoglycaemic episodes per 24 hours compared to SMBG. FlashGM was not associated with decreased time in hypoglycaemia or hyperglycaemia. Based on this systematic review and meta-analysis, there is insufficient evidence to conclude that FlashGM results in a reduction in HbA1_c_ compared to self-monitoring of blood glucose.

The study findings suggest that FlashGM has a stabilising effect on glucose levels, leading to extended time in range and less frequent hypoglycaemic episodes. HbA_1c_ has been considered the gold standard glycaemic outcome that measures blood glucose in the last three months (19). However, it does not account for the intra- and interday glucose levels like time in range does (25–27). Uncontrolled glucose excursions have short- and long-term repercussions for individuals with diabetes (28, 29). FlashGM enables users to have instant access to glucose data with a painless scan. It promotes self-efficacy and increased self-care behaviours as individuals can make insulin and behavioural adjustments in a timely manner (16–18). Thus, FlashGM aids day-to-day glycaemic management as it improves awareness of glucose levels and gives individuals an immediate opportunity to correct glucose values that are outside of the target range (16–18).

### Clinical Implications

FlashGM enables individuals with diabetes to access a comprehensive and personalised glucose profile. Previous studies have demonstrated that an increased frequency of scanning using FlashGM is associated with superior glycaemic management (30–33). Unlike SMBG, individuals do not need to undergo the troublesome procedure of finger-pricking (5). The continuum of glucose data enables people with diabetes to make more informed choices whether it be about food intake, exercise or insulin dosage (16–18). One study showed that participants increased their levels of physical activity after the introduction of FlashGM (16). Easy access to the glucose profile helps to improve self-awareness of exercise levels. Through its data-collecting capacity, FlashGM influences behaviour and encourages individuals to stabilise their glucose levels (16–18, 34).

### Comparison to Other Studies

Previous meta-analyses have reported on the HbA_1c_-reducing effect of FlashGM (7, 9). However, these meta-analyses are susceptible to bias due to their heavy reliance on observational studies (7, 9). Systematic reviews that have included more observational studies tend to report a higher HbA_1c_ reduction. The vast majority of these studies did not have a control group which made them more prone to selection and performance bias. One meta-analysis drew upon the results of 26 observational studies and concluded that the mean change in HbA_1c_ was -0.55% (9). Another systematic review that analysed 10 cohort studies and 3 RCTs reported a -0.51% reduction in HbA_1c_ (7). The meta-analysis on two RCTs and no observational studies reported no difference in HbA_1c_ (8). The inconsistency of findings can be explained by the tendency for observational studies to attribute larger effects than randomised trials (34, 35). In our meta-analysis, we circumvented this limitation by only including RCTs.

Literature on the impact of FlashGM on glycaemic parameters other than HbA_1c_ is lacking. However, there is an increasing body of evidence that supports the use of additional glycaemic metrics (10, 36–38). HbA_1c_ values are skewed in conditions such as pregnancy, anaemia and haemoglobinopathies (26). Unlike HbA_1c_, time in range provides insight into glucose excursions and acute episodes of hypo/hyperglycaemia (10–12, 25–27). Previous studies have demonstrated an association between decreased time in range and the development of microvascular complications (6, 11). Large-scale observational studies supported our findings that FlashGM increases time in range (32, 39, 40). Hence, FlashGM has the potential capacity to reduce harmful glucose fluctuations and to decrease the risk of developing vascular complications.

Previous publications have reported mixed findings on how FlashGM impacts time in hypoglycaemia (7, 29, 40–42). The difference in results can be due to different definitions of hypoglycaemia as some studies set the threshold at <3.9mmol/L (15, 30, 41) whilst others set it at 3.3mmol/L (43). We set the threshold <3.9mmol/L because that is the glucose concentration at which glucose counterregulatory systems are activated (26). Our meta-analysis did not find a significant decrease amongst FlashGM users and this may be attributed to multiple factors. Firstly, past conclusions have been based on observational studies that use baseline data instead of a control group. A major advantage of RCTs is that they minimise performance bias by having both groups wear a glucose sensor. Furthermore, selection bias is reduced through stratified randomisation in the included RCTs. The avoidance of such biases help to explain the findings of this meta-analysis.

Our results showed a statistically significant decrease in frequency of hypoglycaemic episodes after FlashGM was introduced. Past studies have also found an improvement in number of hypoglycaemic episodes experienced by FlashGM users (40, 44). The device equips people with the data to increase their self-awareness of low glucose levels (17). This is evidenced by an observational study that analysed the glucose data of 10,370 users of the Freestyle Libre Flash Glucose Monitoring system (45). The study showed that there was a significant reduction in the Gold Score for hypoglycaemic unawareness after FlashGM use (45). This finding is key to diabetes treatment because hypoglycaemic episodes are linked to severe vascular complications such as myocardial infarction and cardiac arrhythmias (14, 45–47).

We did not find a significant difference in HbA_1c_ or time in hypo/hyperglycaemia after the introduction of FlashGM. One contributing factor could be the initial HbA_1c_ of participants as those who already have optimal glycaemic management are less likely to further decrease their HbA_1c_ (7, 9, 48). The mean baseline HbA_1c_ in this meta-analysis was 64 mmol/mol (7.97%) which is suboptimal but larger reductions in HbA_1c_ would be expected with higher HbA_1c_. Another consideration is patient education. Numerous studies have cited the importance of education on FlashGM and its influence on glycaemic management (34, 49, 50). If individuals are unsure about how to adjust insulin, diet and physical activity to their glucose readings, their ability to self-manage is compromised (49, 50).

Past systematic reviews on continuous glucose monitoring (CGM) have reported its efficacy in reducing HbA_1c_ and increasing time in range (8, 48). It should be noted that these systematic reviews included data from other forms of CGM and only drew findings from two RCTs that focused specifically on FlashGM (8, 48). To our knowledge, this is the first meta-analysis to assess glycaemic outcomes of FlashGM based on data from RCTs. Other systematic reviews have assessed different modes of CGM, which precludes a focused assessment of FlashGM and its impact on glycaemic outcomes. Such reviews have concluded that real-time continuous glucose monitoring (RT-CGM) is superior to FlashGM in glycaemic management (8, 48).

Increased treatment satisfaction (24, 34, 42) and improved quality of life (18, 42) are consistently reported outcomes in study participants that use FlashGM. One cohort study measured the responses of 1913 adults on the Diabetes Treatment Satisfaction Questionnaire (DTSQ) and found that treatment satisfaction improved significantly after introduction of FlashGM (42). Four RCTs in our meta-analysis measured DTSQ scores in its participants (20–22). All four studies reported significant improvements in total treatment satisfaction at follow-up. User questionnaire results show a strong preference for the convenience, flexibility and availability of sensor glucose data (20, 21, 23, 24), thereby highlighting the capacity of FlashGM to improve quality of life for people with diabetes.

### Strengths

The main strengths of this meta-analysis are the exclusive inclusion of RCTs and the assessment of multiple metrics of glycaemic management. The RCTs provide high quality evidence that help guide the fast-growing usage of FlashGM. Such findings may influence decisions about national reimbursement, insurance funding and integration into clinical practice. In this review, we extended our analysis beyond HbA_1c_ and explored other markers of glycaemic status such as time in range and time in hypo/hyperglycaemia. Such outcomes account for day-to-day glucose fluctuations and are increasingly included in interventional studies (12, 25).

### Limitations

This systematic review is limited by the small number of studies which could be included due to its focus on RCTs. The small number of studies precluded subgroup analysis of diabetes type and other variables. Given the inconsistency of different study designs, we found it imperative to prioritise quality of evidence and to only select RCTs. Included studies had an open-label design as participants could access real time glucose readings and accordingly adjust their behaviour. This is characteristic of all continuous glucose monitoring studies as access to sensor-based data is key. Furthermore, this meta-analysis had substantial heterogeneity which could influence the validity of findings. To mitigate this limitation, a random-effects model was used to analyse results.

### Future Directions

The widespread uptake of FlashGM drives the need to evaluate and maximise its efficacy in glycaemic management. When there are more available RCTs, they should be included in future meta-analyses. The longest study in this systematic review is 24 weeks so it remains unclear whether such outcomes will be sustained beyond 24 weeks and for years to come and this is an important question which needs to be addressed. Studies have also started to delve into how FlashGM can be optimised for people with diabetes (49, 50). This includes providing structured educational programs to better inform users (49, 50). Recently, the Food and Drug Administration has approved the Freestyle Libre 2 in the United States (51). The new technology has the capacity to alert patients about high and low glucose levels (51). It would be worthwhile to assess the benefits of these additional features for glycaemic management.

## Conclusions

The findings of this systematic review and meta-analysis demonstrate that flash glucose monitoring does not result in a difference in HbA_1c_. However, its usage leads to improvement in key glycaemic markers, including increased time in range and reduced frequency of hypoglycaemic episodes. To our knowledge, this is the first meta-analysis to delineate the effects of FlashGM on glycaemic management using only randomised controlled trial data. Given a relatively small number of RCTs, further research is needed to determine the impact of FlashGM in the long-term. Through this meta-analysis, we found that flash glucose monitoring has the capacity to improve glycaemic variability and overall clinical care of people with type 1 and type 2 diabetes.

## Data Availability Statement

The original contributions presented in the study are included in the article/**Supplementary Material**. Further inquiries can be directed to the corresponding author.

## Author Contributions

BL conducted the literature search, data extraction, interpreted study findings and wrote the manuscript. DK and SB conducted data analyses, helped with interpretation of study findings and reviewed the manuscript. MH conducted the literature search, data extraction, reviewed and edited the manuscript. NZ helped conduct the systematic review and reviewed the manuscript. EE conceptualised the study, helped with interpretation of study findings, reviewed and edited the manuscript. All authors approved the final version of the manuscript.

## Funding

There was no funding source for this study. EIE’s institutions Austin Health and The University of Melbourne receives research funding from National Health and the Medical Research Council of Australia, Juvenile Diabetes Research Foundation, Sanofi, Novo Nordisk, Eli Lilly, Gilead, Bayer for unrelated research work.

## Conflict of Interest

The authors declare that the research was conducted in the absence of any commercial or financial relationships that could be construed as a potential conflict of interest.

## Publisher’s Note

All claims expressed in this article are solely those of the authors and do not necessarily represent those of their affiliated organizations, or those of the publisher, the editors and the reviewers. Any product that may be evaluated in this article, or claim that may be made by its manufacturer, is not guaranteed or endorsed by the publisher.
